# *TLR1* polymorphisms are significantly associated with the occurrence, presentation and drug-adverse reactions of tuberculosis in Western Chinese adults

**DOI:** 10.18632/oncotarget.23067

**Published:** 2017-12-08

**Authors:** Wu Peng, Hao Chen, Zhenzhen Zhao, Xuejiao Hu, Yi Zhou, Yingyu Li, Lian Yang, Xuemei Wang, Jiajia Song, Tangyuheng Liu, Qian Wu, Hao Bai, Xiaojun Lu, Jie Chen, Binwu Ying

**Affiliations:** ^1^ Department of Laboratory Medicine, West China Hospital, Sichuan University, Chengdu 610041, P.R. China; ^2^ Department of Laboratory Medicine, Chengdu Women and Children's Central Hospital, Chengdu 610091, P.R. China

**Keywords:** tuberculosis, Toll-like receptor 1, single nucleotide polymorphisms, anti-TB drugs, adverse reactions

## Abstract

**Background:**

Obtaining further knowledge regarding single nucleotide polymorphisms in the Toll-like receptor1 gene is of great importance to elucidate immunopathogenesis and management of tuberculosis.

**Results:**

Rs5743565 and rs5743557 were significantly associated with reduced predisposition to TB regarding the mutant allele in additive and dominant models with odds ratios (ORs) ranging from 0.61 to 0.83. There was increased tuberculosis risk associated with the haplotype CAG (rs4833095/rs76600635/rs5743596) [OR (95% CI) = 1.33 (1.07–1.65)] and with haplotype GG (rs56357984/rs5743557) [OR = 1.21 (1.02–1.43)]. The erythrocyte and hemoglobin levels were significantly higher in TB patients with the rs5743557 GG genotype than for AA and/or AG genotype carriers (*p* = 0.006 and 0.020, respectively). The occurrence rates of chronic kidney damage and hepatotoxicity were 21.56% and 10.32%, respectively. Rs5743565 seemed to pose a higher risk of anti-TB-induced hepatotoxicity under the dominant model [OR = 2.17 (1.17–4.05)], and rs76600635 GG/AG genotypes were clearly correlated with the development of thrombocytopenia [OR = 2.98 (1.26–7.09)].

**Conclusions:**

Rs5743565 and rs5743557 in the TLR1 gene may contribute to decreased risk for tuberculosis susceptibility in a Western Chinese population. Rs5743565 and rs76600635 are potential risk factors for adverse reactions to anti-TB drugs.

**Methods:**

We enrolled 646 tuberculosis patients and 475 healthy controls from West China. Six single nucleotide polymorphisms in Toll-like receptor1 gene were genotyped in every individual and were analyzed for their association with tuberculosis susceptibility and clinical presentation. The prospective follow-up was performed to determine whether these single nucleotide polymorphisms are associated with adverse reactions to anti-tuberculosis drugs.

## INTRODUCTION

Tuberculosis (TB), caused by pathogenic *Mycobacterium tuberculosis* (MTB), remains a significant challenge to global public health [1]. According to the World Health Organization (WHO) report released in 2016, there were an estimated 10.4 million newly diagnosed tuberculosis cases in 2015 worldwide [2]. TB not only impacts people's health but also leads to huge economic burdens to the individual's family and society at-large [3]. A third of the global population is thought to be infected with the MTB strain, however, the majority of infections remain latent, without clinical symptoms and signs associated with active TB [4]. Accumulating data have demonstrated that host genetic background components can influence the occurrence, progression, and clinical outcome of TB infection [5–6]. Many research efforts have focused on identifying host genomic variants that are significantly associated with TB development as well as clinical phenotypes [7–8]. A large GWAS study from Sveinbjornsson G *et al.* showed that HLA class II variants (rs557011, rs9271378 and rs9272785) contribute to the complex genetic risk of tuberculosis, possibly through reduced presentation of protective MTB antigens to T cells [9]. A study by Hu *et al.* revealed that tuberculosis patients carrying TT genotypes of rs4736958 and rs7832767, both within the *SFRP1* gene, correlate with higher C-reactive protein (CRP) concentrations compared with other genotypes [8]. A better understanding of genetic predisposition to TB would result in improved development of novel approaches for TB prevention and treatment, and contribute to unravelling the pathogenesis of this disease. However, considering the limited risk genetic variants and their finite significance on TB susceptibility, the exact host genetic elements that are involved in the biological process of TB infection remain to be understood.

The host innate immune response plays a pivotal role in defense against invading pathogens, through contact of the host pattern recognition receptors (PRRs) with pathogen-associated molecular patterns (PAMPs), which are defined as conserved structures within the cell wall or genetic components of bacteria [10–11]. Thus far, members of the Toll-like receptor (TLR) family are the most characterized PRRs in human. Importantly, Toll-like receptor 1 (TLR1), a member of the TLR family, is responsible for recognition of several MTB antigens by forming a heterodimer with Toll-like receptor 2 molecule (TLR2) [12–13]. For instance, Shin DM *et al*. revealed that mycobacterial lipoprotein LpqH induces a TLR2/1/CD14 cascade and robustly contributes to effective anti-bacterial autophagy through vitamin D receptor signaling activation [13]. As the TLR1 molecule participants in the process of TB infection, polymorphisms within the *TLR1* gene have the potential to influence genetic predisposition to TB disease by causing structural and/or functional alterations in this receptor, subsequently leading to altered defense responses to MTB invasion.

Previous association studies have yielded several candidate single nucleotide polymorphisms (SNPs) located in the *TLR1* gene that may be associated with the risk of TB infection, however, the results derived from these studies are contradictory and do not allow definitive conclusions [14–16, 20]. Studies have reported that *TLR1* rs5743618 (I602S, T1805G) is a functional genetic polymorphism that possibly influences the susceptibility to TB in certain ethnic groups [14–16]. Ocejo-Vinyals *et al*. reported that the G allele and GG genotype of rs5743618 correlated with an increased risk for pulmonary tuberculosis in a Spanish population [15], and Qi H *et al.* also found that Toll-like receptor 1(TLR1) Gene SNP rs5743618 is associated with increased risk for tuberculosis in Han Chinese children [17]. Nevertheless, Ma *et al*. found no association between rs5743618 and TB infection in a Han Chinese population [16]. Another well-characterized missense variant within the *TLR1* gene, rs4833095, has been shown to be statistically associated with altered risk for IgA nephropathy in children [18], mortality in gram-positive sepsis [19] and prostate cancer development [20]. In addition, allele A of rs4833095 was correlated with reduced susceptibility to TB in an Indian cohort analysis [21]. The work evaluating the relationship between *TLR1* polymorphisms and TB susceptibility has been extensively conducted in Spanish, Indian, and African-American populations. However, due to the heterogeneity of the various populations and divergent genetic backgrounds, whether *TLR1* SNPs affect TB occurrence in Chinese populations is yet to be confirmed. Knowledge about more *TLR1* SNP loci would be important to provide new insight into the immunopathogenesis of TB.

During the clinical management of TB, multiple anti-TB drugs (ATDs) are used, and adverse reactions frequently occur, sometimes contributing to treatment interruption and even therapy failure or mortality. These reactions mainly involve liver injury, kidney damage, or hematological toxicity. Currently, ATD-induced hepatotoxicity (ATDH) has been reported to be the most common adverse response to TB therapies employing isoniazid, rifampicin and pyrazinamide [22]. Several ATDH-susceptible genetic loci were found using genetic association approaches, for example *NAT2* slow acetylation genotypes [23], rs1695 within the *GSTP1* gene [24]. However, previous studies have mainly focused on the clinical profile and associated genetic risk factors of ATDH; little is known regarding the clinical and pharmacogenetic features of other adverse effects induced by ATDs. On the other hand, previous association studies with ATD-induced adverse events are predominantly enriched for drug metabolizing enzyme genes [23–25]. To our knowledge, the potential relationships between *TLR1* gene variations and drug adverse reactions during TB therapy have not been reported and are worth being investigated.

China ranks third among the 22 countries highest-burdened with TB in the world, with the highest annual number of cases of multidrug-resistant tuberculosis (MDR-TB). To provide evidence for the effect of *TLR1* genetic polymorphisms on the development and progression of TB disease, we evaluated the association of *TLR1* SNPs with predisposition to TB diseases, clinical phenotypes and ATD-induced adverse reactions in a prospective cohort of 1,121 adult participants from Western China.

## RESULTS

### Baseline features of the cohort subjects

Table 1 presents the clinical data and laboratory results of 646 cases and 475 controls. No difference was found in age or sex between TB patients and normal controls (*p* = 0.366, 0.176, respectively). For the peripheral complete blood cell count, TB patients exhibited a clear increase in the counts of leucocytes, neutrophils, monocytes and platelets, whereas cases with active TB had reduced levels of erythrocytes, hemoglobin, and lymphocytes (all *p* less than 0.001). As expected, the erythrocyte sedimentation rate (ESR) and the C-reactive protein (CRP) levels in the TB group were significantly higher than the normal levels.

**Table 1:** General characteristics of the study cases and controls

Characteristics	TB^a^ (*n* = 646)	HC^b^ (*n* = 475)	*P*
**Demographic data**			
Gender (males) *n* (%)	394 (61.00)	270 (56.84)	0.176
Age (years)	42.28 ± 19.52	43.21 ± 12.69	0.366
**Laboratory examinations**			
Erythrocytes (×10^12^/L)	4.15 ± 0.84	4.80 ± 0.48	**<0.001**
Hemoglobin (g/L)	116.62 ± 24.56	145.59 ± 15.16	**<0.001**
Hematocrit (%)	0.63 ± 4.88	0.45 ± 0.042	0.435
Leucocytes (×10^9^/L)	7.93 ± 16.75	6.07 ± 1.26	**<0.001**
Neutrophils (×10^9^/L)	5.29 ± 3.76	3.64 ± 0.95	**<0.001**
Lymphocytes (×10^9^/L)	1.27 ± 1.84	1.93 ± 0.76	**<0.001**
Monocytes (×10^9^/L)	0.51 ± 0.36	0.37 ± 0.21	**<0.001**
Platelets (×10^9^/L)	250.40 ± 68.10	170.92 ± 46.89	**<0.001**
ESR (mm/h)	50.00 (25.00–80.00)	-	-
C-reactive protein (mg/L)	28.45 (7.75–76.20)	-	-
Positive smear *n* (%)	170/ 565 (30.09)	-	-
Positive TB-DNA *n* (%)	224/442 (50.68)	-	-
Positive culture *n* (%)	29/160 (18.13)	-	-
**TB clinical subforms *n* (%)**			
PTB^c^	360 (55.73)		
EPTB^d^	142 (21.98)		
PTB & EPTB^e^	144 (22.29)		
**Clinical symptoms on admission**			
Fever *n* (%)	345 (53.41)	-	-
Loss weight *n* (%)	222 (34.37)	-	-
Night sweat *n* (%)	191 (29.57)	-	-
Poor appetite *n* (%)	245 (37.93)	-	-
Fatigue *n* (%)	173 (26.78)	-	-
Adverse reactions to ATDs			
Anemia *n* (%)	40 (9.17)		
Leukopenia *n* (%)	10 (2.29)		
Thrombocytopenia *n* (%)	18 (4.13)		
Hepatotoxicity *n* (%)	45 (10.32)		
Chronic kidney damage *n* (%)	94 (21.56)		

Annotation: TB = tuberculosis; HC = healthy controls; PTB = pulmonary tuberculosis; EPTB = extra-pulmonary tuberculosis; PTB & EPTB = pulmonary tuberculosis combined with extra-pulmonary tuberculosis.

As shown in Table 1, 360 (55.73%) pulmonary tuberculosis (PTB) patients, 142 (21.98%) extra-pulmonary tuberculosis (EPTB), and 144 PTB (22.29%) combined with EPTB (PTB&EPTB) were enrolled in the study. Among these cases, the TB-DNA-positive proportion was 50.68% (224/442), which was higher than the smear and culture positive rates [30.09% (170/565), and 18.13% (29/160), respectively]. Most TB patients in our cohort suffered from fever (53.41%) among those common clinical manifestations. The 436 active TB cases were treated with combinatorial oral-drug chemotherapy, including at least RIF and INH. These eligible cases received regular laboratory tests (biochemical, hematological and urine examinations) once a week in hospital, and subsequently were monitored monthly for 6 months or until the end of treatment. In our analyses, chronic kidney damage (21.56%, 94/436) was the most prevalent side-effect of the RIF and INH regimen, and the second was hepatotoxicity (10.32%, 45/436).

### Correlation of TB susceptibility and SNPs within *TLR1* gene

Genotyping of all six genetic variants was successful for all 646 cases and 475 controls, and the genotype frequency distributions for all 6 SNPs investigated were in line with Hardy-Weinberg ratios in the control group (*p* all >0.05, Supplementary Table 1). The genotyping results of detected SNPs are depicted in Table 2. Statistical differences in allelic and genotypic distributions of rs5743565 and rs5743557 were observed between the TB group and control group (*p all* < 0.05). For rs5743565, the frequency of the C allele was 40.09% in TB cases and 46.42% in the controls, with an estimated OR of 0.77 (95% CI = 0.65–0.91), which suggested that the C allele at this locus may be associated decreased susceptibility to TB development. In rs5743557, the rate of the minor allele (A allele) was 41.18% in TB patients and was 45.68% in normal individuals. The allelic discrepancy was estimated with an OR of 0.83, (95% CI = 0.70–0.99), indicating that the rs5743557 A allele might be correlated with a slight reduction in TB risk.

**Table 2:** Association between genetic polymorphisms in *TLR1* gene and TB risk

SNP	Variant	Case *n* (%)	Control *n* (%)	OR (95% CI)	*P*
rs4833095	T	508 (39.32)	390 (41.05)	0.93 (0.78–1.10)	0.408
C>T	C	784 (60.68)	560 (58.95)		
	TT	102 (15.79)	89 (18.74)		0.414
	TC	304 (47.06)	212 (44.63)		
	CC	240 (37.15)	174 (36.63)		
rs76600635	G	112 (8.669)	98 (10.32)	0.83 (0.62–1.10)	0.186
A>G	A	1180 (91.331)	852 (89.68)		
	GG	4 (0.62)	4 (0.84)		0.409
	GA	104 (16.10)	90 (18.95)		
	AA	538 (83.28)	381 (80.21)		
rs5743596	A	390 (30.19)	297 (31.26)	0.95 (0.79–1.14)	0.585
G>A	G	902 (69.81)	653 (68.74)		
	AA	64 (9.91)	45 (9.47)		0.598
	AG	262 (40.56)	207 (43.58)		
	GG	320 (49.54)	223 (46.95)		
rs5743565	C	518 (40.09)	441 (46.42)	**0.77 (0.65–0.91)**	**0.003**
T>C	T	774 (59.91)	509 (53.58)		
	CC	104 (16.10)	96 (20.21)		**0.007**
	CT	310 (47.99)	249 (52.42)		
	TT	232 (35.91)	130 (27.37)		
rs56357984	A	419 (32.43)	332 (34.95)	0.89 (0.75–1.07)	0.212
G>A	G	873 (67.57)	618 (65.05)		
	AA	69 (10.68)	53 (11.16)		0.307
	AG	281 (43.50)	226 (47.58)		
	GG	296 (45.82)	196 (41.26)		
rs5743557	A	532 (41.18)	434 (45.68)	**0.83 (0.70–0.99)**	**0.033**
G>A	G	760 (58.82)	516 (54.32)		
	AA	116 (17.96)	93 (19.58)		**0.032**
	AG	300 (46.44)	248 (52.21)		
	GG	230 (35.60)	134 (28.21)		

Annotation: *p* value was calculated using logistic regression analysis.

In this study, three genetic patterns, including additive, dominant, and recessive patterns, were constructed to further explore differences in the distributions of SNP genotypes. The results of heritance model analysis are displayed in Table 3. The statistical significances of the additive and dominant patterns for rs5743565 were determined. For the rs5743565 locus, under the additive model (CC *versus.* TT), the CC genotypes seemed to occur at a higher rate in samples free from active TB, with an estimated OR-value of 0.61 (*p* = 0.005), and subjects carrying the CC and/or CT genotype showed a 0.67-fold lower risk for TB infection compared to those with the TT genotype when the dominant model was applied (CC+CT *versus.* TT: OR = 0.67, 95% CI = 0.52–0.87, *p* = 0.003). Analogously, rs5743557 showed a statistical association with reduced predisposition to TB under the dominant model (AA+AG *versus.* GG: *p* = 0.009, OR = 0.71, 95% CI = 0.55–0.92). No statistically significant correlations were observed with regard to the genotype, allelic frequencies and three genetic models of the remaining four SNPs (rs4833095, rs76600635, rs5743596, and rs56357984) between the two groups (all *p* > 0.05).

**Table 3:** Analysis of *TLR1* genetic variants relevant to TB risk in a Han Chinese population

SNP	Additive model		Dominant Model		Recessive Model	
	OR (95% CI)	P^*^	OR (95% CI)	P^*^	OR (95% CI)	P^*^
rs4833095 C>T	0.93 (0.78–1.10)	0.408	0.98 (0.77–1.25)	0.859	0.81 (0.59–1.11)	0.195
rs76600635 A>G	0.83 (0.62–1.10)	0.188	0.81 (0.60–1.11)	0.187	0.73 (0.18–2.95)	0.663
rs5743596 G>A	0.95 (0.79–1.14)	0.610	0.90 (0.71–1.14)	0.392	1.05 (0.70–1.57)	0.809
rs5743565 T>C	**0.61 (0.43–0.86)**	**0.005**	**0.67 (0.52–0.87)**	**0.003**	0.76 (0.56–1.03)	0.076
rs56357984 G>A	0.89 (0.75–1.07)	0.222	0.83 (0.65–1.06)	0.129	0.95 (0.65–1.39)	0.80
rs5743557 G>A	0.73 (0.51–1.03)	0.070	**0.71 (0.55–0.92)**	**0.009**	0.90 (0.66–1.22)	0.491

Annotation: *p* value was calculated using logistic regression analysis.

### Association of two TB-related SNPs within the *TLR1* gene with pulmonary tuberculosis risk

We subsequently analyzed whether the two TB-related variants (rs5742565 and rs5743557) were associated with pulmonary tuberculosis, the main clinical subtype of TB in our cohort. As shown in Table 4, the allelic distribution of rs5743565 was obviously distinct between the pulmonary TB subgroup and control, with a *p*-value of 0.035 and OR = 0.81. Regarding the genotypic distribution of rs5743565, significant difference was found only in the dominant model in sub-group analysis (CC+CT *versus.* TT: OR = 0.71, 95% CI = 0.53–0.95, *p* = 0.022). For variation rs5743557, the A alleles (AA and/or AG genotype) were correlated with a decreased risk for pulmonary tuberculosis compared with those with homozygous GG genotype under the dominant pattern (*p* = 0.036, OR = 0.73, 95% CI = 0.54–0.98). These results indicated that the rs5743565 and rs5743557 alleles seemed to have a weak protective effect against pulmonary TB compared with all forms of active TB.

**Table 4:** Association between 2 SNPs within *TLR1* Gene and pulmonary TB risk

SNP	Variant	PTB *n* (%)	Control *n* (%)	OR (95% CI)	*P*
rs5743565	C	297 (41.25)	441 (46.42)	**0.81 (0.67–0.99)**	**0.035**
T>C	T	423 (5.75)	509 (53.58)		
	CC	62 (17.22)	96 (20.21)		0.068
	CT	173 (48.06)	249 (52.42)		
	TT	125 (34.72)	130 (27.37)		
Additive	CC	62 (17.22)	96 (20.21)	0.67 (0.45–1.00)	0.052
	TT	125 (34.72)	130 (27.37)		
Dominant	CC+CT	235 (65.28)	345 (72.63)	**0.71 (0.53–.95)**	**0.022**
	TT	125(34.72)	130 (27.37)		
Recessive	CC	62 (17.22)	96 (20.21)	0.82 (0.58–1.17)	0.275
	CC+CT	298 (82.78)	379 (79.79)		
rs5743557	A	296 (41.11)	434 (45.68)	0.83 (0.68–1.01)	0.062
G>A	G	424 (58.89)	516 (54.32)		
	AA	62 (17.22)	93 (19.58)		0.108
	AG	172 (47.78)	248 (52.21)		
	GG	126 (35.00)	134 (28.21)		
Additive	AA	62 (17.22)	93 (19.58)	0.71 (0.47–1.06)	0.094
	GG	126 (35.00)	134 (28.21)		
Dominant	AA+AG	234 (65.00)	341(71.79)	**0.73 (0.54–0.98)**	**0.036**
	GG	126 (35.00)	134 (28.21)		
Recessive	AA	62 (17.22)	93 (19.58)	0.86 (0.60–1.22)	0.386
	AG+GG	298 (82.78)	382 (80.42)		

Annotation: *p* value was calculated using Chi-square test.

### Haplotype analysis

**Figure 1:**
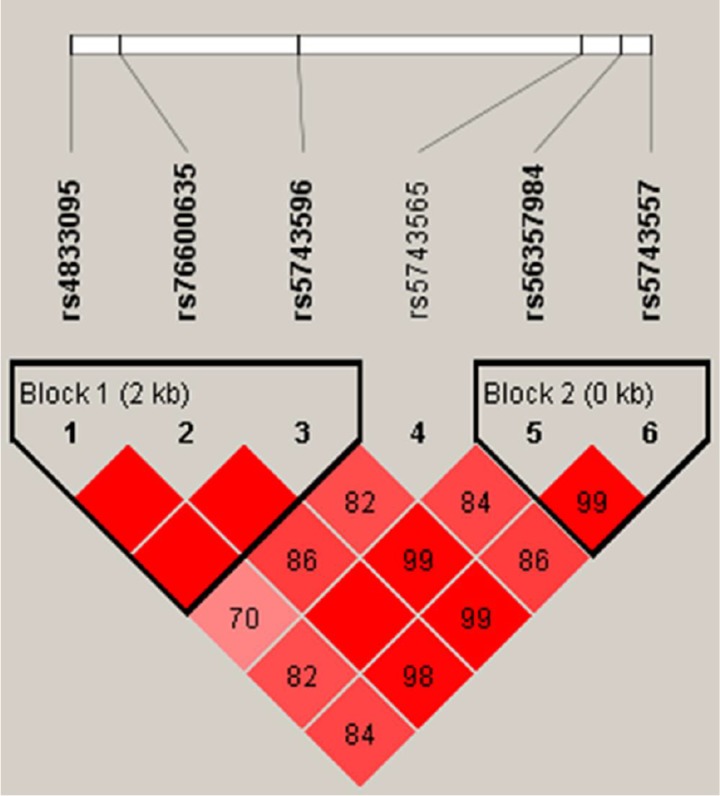
Linkage disequilibrium (LD) structures of SNPs in the *TLR1* gene LD between all pairs of SNPs was evaluated by D′-statistics. The D′-values (%) are presented in the squares. Pairwise D′-values are color coded: high D′-values are dark and low D′-values are light.

We performed haplotype analyses for *TLR1* genetic polymorphisms, and two haplotype block sets were identified, as shown in Figure 1. The SNPs rs4833095, rs76600635 and rs5743596 were in strong linkage disequilibrium (LD) with a coefficient D′ > 0.99, and made up the block 1 haplotype. The other haplotype block was comprised of rs56357984 and rs5743557 (D′ > 0.99). Table 5 shows the two haplotype frequencies in TB cases and controls as well as their associations with tuberculosis disease risk. The haplotype CAG from rs4833095, rs76600635 and rs5743596 was associated with significantly increased tuberculosis susceptibility at a *p*-value of 0.009 (OR = 1.33, 95%; CI = 1.07–1.65). Another haplotype GG (within rs56357984 and rs5743557) was statistically associated with an increased TB risk (*p* = 0.029, OR = 1.21, 95% CI = 1.02–1.43), which was consisten with the SNP rs5743557 individual association results.

**Table 5:** Haplotype association of the *TLR1* variants with the risk of TB

Haplotype	Freq	Case ratios	Control ratios	*p*-value	OR (95% CI)
**Block 1**					
TAG	0.401	508:784	390:560	0.408	0.93 (0.78–1.10)
CAA	0.306	390:902	297:653	0.585	0.95 (0.79–1.14)
CAG	0.199	282:1010	165:785	**0.009**	**1.33 (1.07–1.65)**
CGG	0.094	112:1180	98:852	0.186	0.83 (0.62–1.10)
**Block 2**					
GG	0.569	759:531	514:434	**0.02**9	**1.21 (1.02–1.43)**
AA	0.334	417:873	331:617	0.199	0.89 (0.75–1.06)
GA	0.097	114:1176	103:845	0.109	0.80 (0.60–1.05)

Annotation: *p*-value was calculated using Chi-square test.

Block 1 consisted of rs4833095, rs76600635 and rs5743596 loci.

Block 2 consisted of SNPs rs56357984 and rs5743557

### Relationship of TB clinical features with significant SNPs

We have previously shown that a specific genetic variant was able to influence the clinical phenotypes of active tuberculosis [8]. Therefore, we evaluated the potential relationship between two discrepant SNPs (rs5743565 and rs5743557) and several common TB disease presentations on admission and stratified patients based on the polymorphism loci genotypes. In general, the clinical characterization of TB patients was similar between these two SNPs as shown in Tables 6 and 7. In rs5743557, the levels of erythrocytes and hemoglobin were significantly higher in TB patients with the GG genotype compared with the AA and AG genotype carriers (*p* = 0.006 and 0.020, respectively, Table 7). None statistical association of TB clinical parameters with different rs5743565 genotypes was found.

**Table 6:** Association of the rs5743565 polymorphism with clinical traits of TB patients

Characterizations	CC (*N* = 104)	CT (*N* = 310)	TT (*N* = 232)	*P*
**General information**				
Age (years)	40.49 ± 17.83	42.53 ± 19.77	42.75 ± 19.96	0.591
Gender (male/female)	54/50 (51.92)	192/118 (61.94)	151/81 (65.09)	0.070
Smoking (yes/no)	80/24 (76.92)	217/93 (70.00)	155/77 (66.81)	0.174
Alcohol (yes/no)	82/22 (78.85)	220/90 (70.97)	169/63 (72.84)	0.294
**Manifestations *n* (%)**				
Fever	44 (42.31)	130 (19.35)	104 (44.83)	0.787
Weight loss	75 (72.12)	195 (62.90)	146 (62.93)	0.200
Night sweat	78 (75.00)	213 (68.71)	155 (66.81)	0.319
Poor appetite	64 (61.54)	184 (31.80)	145 (62.50)	0.750
Fatigue	73 (70.19)	219 (70.65)	172 (74.14)	0.480
**Laboratory examinations**				
Erythrocyte (×10^12^/L)	4.09 ± 0.86	4.11 ± 0.84	4.23 ± 0.82	0.177
Hemoglobin (g/L)	113.52 ± 27.52	115.99 ± 23.97	118.85 ± 23.84	0.153
WBC (×10^9^/L)	8.71 ± 22.79	7.20 ± 4.00	8.57 ± 22.99	0.563
Platelets (×10^9^/L)	229.99 ± 108.17	257.70 ± 147.88	251.93 ± 113.69	0.173
C-reactive protein (mg/L)	21.80 (8.10–62.70)	15.85 (1.98–67.93)	15.00 (1.93–64.35)	0.264
ESR (mm/h)	40.00 (20.50–73.50)	43.50 (15.00–77.25)	41.00 (14.00–75.00)	0.389

**Table 7:** Association of rs5743557 polymorphism with clinical traits of patients with TB

Characterizations	AA (*N* = 116)	AG (*N* = 300)	GG (*N* = 230)	*P*
**General information**				
Age	41.71 ± 18.35	42.42 ±19.91	42.38 ± 19.67	0.941
Gender (male/female)	62/54 (53.45)	189/111 (63.00)	143/87 (62.17)	0.181
Smoking *n* (%)	86/30 (74.14)	203/97 (67.67)	163/67 (70.87)	0.406
Alcohol *n* (%)	86/30 (74.14)	214/86 (71.33)	171/59 (74.35)	0.702
**Manifestations *n* (%)**				
Fever	50 (43.10)	125 (41.67)	103 (44.78)	0.773
Weight loss	82 (70.69)	195 (65.00)	139 (60.43)	0.894
Night sweat	83 (71.55)	206 (68.67)	157 (68.26)	0.808
Poor appetite	74 (63.79)	184 (61.33)	135 (58.70)	0.638
Fatigue	81 (69.83)	220 (73.33)	163 (70.87)	0.715
**Laboratory examinations**				
Erythrocyte (×1012/L)	4.06 ± 0.80	4.08 ± 0.81	4.29 ± 0.87	**0.00s6**
Hemoglobin (g/L)	114.69 ± 22.74	114.58 ± 25.09	120.26 ± 24.44	0.020
WBC (×109/L)	8.80 ± 21.65	7.17 ± 3.88	8.49 ± 23.09	0.556
Platelets (×109/L)	231.61 ± 108.93	256.20 ± 122.89	254.47 ± 148.89	0.203
C-reactive protein (mg/L)	14.20 (1.49–54.23)	15.90 (2.16–68.90)	13.75 (1.83–62.18)	0.567
ESR (mm/h)	37.50 (5.25–77.75)	48.00 (15.00–79.00)	43.50 (15.00–77.00)	0.259

### Relationships between 6 *TLR1* SNPs and adverse drug reactions from TB treatment

We assessed whether six SNPs in *TLR1* gene were potentially associated with five types of adverse reactions to ATD. Unfortunately, we were unable to identify a statistical correlation between the 5 kinds ATD adverse responses and loci rs4833095, rs5743596, rs56357984, and rs5743557 (Supplementary Tables 2–5). Rs5743565 was shown to be significantly associated with the occurrence of ATDH under the dominant model. As shown in Table 8, TB patients with the TT genotype were more likely to suffer from hepatotoxicity to ATDs compared to patients carrying CC/CT genotypes (TT: 15.33%, *versus.* CC+CT: 7.69%; *p* = 0.013, OR = 2.17, 95% CI = 1.17–4.05). Furthermore, we identified a significant link between rs76600635 and ATD-associated thrombocytopenia, with a *p*-value of 0.010. The proportion of thrombocytopenia in patients with GG+AG genotypes was 11.54%, whereas it was 4.19% in AA carriers, with an OR of 2.98, and 95% CI ranging from 1.26 to 7.09 (Table 9).

**Table 8:** Association of rs5743565 with ATD adverse drug reactions

Drug adverse reactions	TT (*n* = 150)	CC+CT (*n* = 286)	*P*	OR (95% CI)
**Anemia *n* (%)**	12 (8.00)	28 (9.79)	0.538	0.80 (0.39–1.63)
**Leukopenia *n* (%)**	4 (2.67)	6 (2.10)	0.968	1.28 (0.36–4.6)
**Thrombocytopenia *n* (%)**	4 (2.67)	14 (4.90)	0.267	0.53 (0.17–1.65)
**Hepatotoxicity (%)**	23 (15.33)	22 (7.69)	**0.013**	**2.17 (1.17–4.05)**
**Chronic kidney damage *n* (%)**	34 (22.67)	60 (20.98)	0.684	1.10 (0.69–1.78)

Annotation: *p*-value was calculated using Chi-square test after adjusting with age and gender

**Table 9:** Association of rs76600635 with ATD adverse drug reactions

Drug adverse reactions	GG+AG (*n* = 78)	AA (*n* = 358)	*P*	OR (95% CI)
**Anemia *n* (%)**	4 (5.13)	36 (10.06)	0.172	0.48 (0.17–1.4)
**Leukopenia *n* (%)**	1 (1.28)	9 (2.51)	0.809	0.50 (0.06–4.03)
**Thrombocytopenia *n* (%)**	9 (11.54)	15 (4.19)	**0.010**	**2.98 (1.26–7.09)**
**Hepatotoxicity (%)**	6 (7.69)	39 (10.89)	0.400	0.68 (0.28–1.67)
**Chronic kidney damage *n* (%)**	17 (21.79)	77 (21.51)	0.956	1.02 (0.56–1.84)

Annotation: *p*-value was calculated using Chi-square test after adjusting with age and gender.

## DISCUSSION

TLR1 cooperates with TLR2 and mediates the important innate immune recognition of lipoproteins that are widely present in MTB strains. Several TB-associated *TLR1* SNPs have been identified in numerous diverse ethnic populations, for example rs4833095 [21] and rs5743618 [15–16]. Motivated by these prior works, the present study further assessed correlations between common genetic variants in *TLR1* and TB in a Western Chinese population. The results indicated that *TLR1* rs5743565 and rs5743557 may contribute to a genetic risk for TB infection in a Western Chinese population. Rs5743565 and rs76600635 were observed to be significantly associated with the occurrence of ATDH and thrombocytopenia, respectively.

The C allele of rs5743565 was found to be a promising protective factor for tuberculosis susceptibility in a Western Chinese population. A similar role in immunologic protection at this locus was demonstrated in a paper by Xiao W *et al*, which indicated that rs5743565 contributed to a declined risk for Graves’ disease in a Chinese Cantonese population [28]. We also discovered that the rs5743557 A allele appeared to have a slight protective effect for TB infection. Published studies have shown that rs5743557, in the *TLR1* promoter region, is not associated with the development of IgA nephropathy in a Chinese Han population [29] or benign prostatic hyperplasia in a Korean population [30], whereas it is significantly correlated with a high risk for childhood IgA nephropathy in a Korean population [18]. The data described above strongly indicate that various SNPs have different impacts on the development and progression of human disorders depending on iverse pathological conditions and ethnicity. Nevertheless, there is very little information about the association of rs5743565 and rs5743557 with infectious diseases, and further studies from a wide range of ethnic and racial populations are required to confirm the predisposition risk related to these two SNPs. According to the bioinformatics software SNPinfo software (http://snpinfo.niehs.nih.gov/), both rs5743565 and rs5743557 are predicted to lie within a transcription factor binding site (TFBS), and they might potentially influence the binding of transcription factors and cause alterations in *TLR1* gene expression, thus disregulating the immune signaling cascade and multiple inflammatory genes, and finally contributing to abnormal host immune response to MTB infection. In addition to transcription factor conjugation, other potential mechanisms underlying these two variants associated with TB protection are worth to be explored, for example DNA methylation epigenetics and long non-coding RNA. Mechanism studies are needed to confirm the above speculation.

It has been reported that *TLR1* SNP rs4833095 associated with the protection for TB disease. In addition, the next functional studies using transfected HEK cells with rs4833095-AA (or TT) genotype showed an increased NF-kB induction after stimulation with MTB [21]. However, our results failed to gain the significant correlation of TB susceptibility and rs483095 variant. The haplotype CAG, consisting by rs4833095, rs76600635 and rs5743596, were significant risk factor for TB infection with OR-value of 1.33. The cumulative effect causing by multiple SNPs may in part explain the difference in individual SNP and haplotype analyses. In addition, these findings implicate that T allele in rs4833095 locus seem to be the favorable event for this disease which are its precise function is pressingly warranted to verify in the larger cohort analysis. Another haplotype GG, comprised of rs56357984 and rs5743557, was related to the increase in the risk for TB, which was consistent with the individual rs5743557 association results. In this study, we characterized the relationship between TB clinical parameters and two discrepant SNPs. For rs5743557, TB patients with homozygous GG genotype were more likely to have greater levels of erythrocyte and hemoglobin than AA/AG carriers. For rs5743565, none statistical link with TB clinical features was found.

Though this study found that rs5743565 and rs5743557 reduce the risk of tuberculosis in western China population, these two SNPs could only partially explain why TLR1 affects the pathogenesis of tuberculosis. The relationship between TLR1 gene polymorphism and the incidence of tuberculosis is also associated with other SNPs or other biological mechanisms. For example, Johnson CM *et al*. report that I602S(rs5743618), a common single nucleotide polymorphism within TLR1, is associated with aberrant trafficking of the receptor to the cell surface and diminished responses of blood monocytes to bacterial agonists [31]. Similarly, the observations of Hawn TR *et al*.'s demonstrate that variation in the inflammatory response to bacterial lipopeptides caused by I602S(rs5743618) is regulated by a common TLR1 transmembrane domain polymorphism [32]. Additionally, Uciechowski P *et al.* found that Lack of TLR1 surface expression accompanied by impaired function was strongly associated with TLR1 SNP G743A(rs202131936) [33]. Therefore, on condition that we intend to interpret the role of TLR1 in the pathogenesis of tuberculosis accurately, we should understand the changes of more SNPs comprehensively. Our research supplemented data for this progress.

No association of the other 4 SNPs(rs5743596, rs4833095, rs76600635, rs56357984) was found with tuberculosis in this study. For rs5743596 and rs4833095, our study results were consistent with Hui Qi *et al*'s [17]. Interestingly, other researches found significant relationship between rs4833095 and tuberculosis [34–36]. Diversities in racial, environmental and tuberculosis prevalence may account for this discrepancy. With regard to rs76600635 and rs56357984, TB-related research was very few to date. These two SNPs may be unrelated with the risk of tuberculosis in western China. It was also possible that we did not detect the difference between case and control effectively because of the sample size. They could be interrogated in multicenter studies with large samples in later research.

During the process of tuberculosis clinical management, adverse drug reactions owing to anti-TB chemotherapy are very important monitoring program. Better understanding of the risk factors for anti-TB-induced adverse reactions may be useful to prevent their occurrence and are favorable to treatment completion. We comprehensively investigated multiple adverse responses from ATDs in the present study and further evaluated their associations with these 6 SNPs in *TLR1* gene. Previous investigators have showed several risk genetic biomarkers for hepatotoxicity from TB treatment [22–24]. For the first time, we identified that *TLR1* rs5743565 might perform as a predisposing risk factor, and TT genotypes carriers were nearly 2.17-time more likely to develop hepatotoxicity based on rifampicin and isoniazid anti-TB compared with patients having CC/CT genotypes. Although roles of *TLR1* gene on drug adverse reactions to ATDs remain unclear, our findings provide the useful cues for the further research. In the rs7660635 locus, we surprisingly found that GG+GA carriers had a 2.98-fold increase in the risk for the occurrence of thrombocytopenia from ATDs than those with AA genotype. Thrombocytopenia rarely occurs during the TB chemotherapy (the rate was 4.13% in the present study), but its manifestations were serious and clinical outcome was poor. Pereira J *et al* characterized that rifampicin-induced thrombocytopenia belonged to the immunological reaction, by which the binding of the rifampicin-dependent antibody to platelets [37]. However, the thrombocytopenia caused by isoniazid was only reported in several clinical cases [38] and the mechanisms underlying remains unclearly. The rs7660635 SNP may potentially behavior as the predictor for the thrombocytopenia to ATDs and require the large cohort validation in the future.

Some limitations in our study were included. First, the relatively small sample size in our cohort may restrict the power to recognize some trivial associations, which should be interrogated in multicenter studies with large samples. Second, because of the limited space, the gene-gene and gene-environment interactions did not been performed in this study. Furthermore, functional exploration inferring these candidate SNPs should be conducted further to confirm our findings.

In conclusion, SNP rs5743565 C allele and rs5743557 A allele within TLR1 gene were significantly associated with the reduced risk for tuberculosis in Western Chinese population. Rs5743565 was associated with the occurrence of anti-TB induced hepatotoxicity, and rs76600635 significantly correlated with the development of thrombocytopenia, suggesting these SNPs may be genetic biomarker for the development and progression of TB disease.

## MATERIALS AND METHODS

### Study participants

In total, 1121 adult participants from West China Hospital of Sichuan University were enrolled in the study between December 2014 and November 2016, including 646 patients with tuberculosis and 475 healthy controls. Eligible cases were individuals with active TB disease diagnosed by at least two independent, experienced specialists based on bacteriological evidence (positive smear/culture/TB-DNA result), TB-related radiological findings and appropriate responses to anti-TB chemotherapy. The exclusion criteria for patients included HIV/HBV/HCV-serological positive results, primary immunodeficiency, immunosuppression therapy, other infectious diseases, cancers, pregnancy, and autoimmune diseases. The 475 normal control individuals, who had normal physical examination results and were free from past TB infection, were recruited from the cohort of the physical examination population. All subjects in this study were unrelated Han Chinese from West China, and the control group was frequency-matched with the case group regarding age and gender. For each studied subject, demographic and clinical information was extracted from the medical record system.

**Figure 2:**
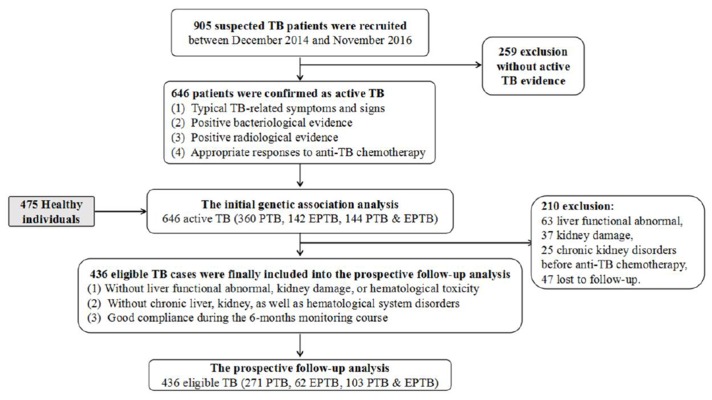
The workflow design of our study Two parts were included in our analysis: the first case-control study and the subsequent follow-up analysis.

Subsequently, we conducted a prospective follow-up study to evaluate whether SNPs within the *TLR1* gene might potentially be associated with adverse reactions to anti-TB drugs (ATDs). Anti-TB chemotherapy regimens employed in our analysis involved at least isoniazid (INH, 300–400 daily) and rifampicin (RIF, 450–600 daily) for 6 months or longer. Among the 646 cases with active TB disease, only 436 eligible patients were finally included into the follow-up study. The conditions for eligible cases were as follows: (1) free from liver function abnormalities, kidney damage, or hematological toxicity before anti-TB chemotherapy; (2) without chronic liver, kidney, or hematological system disorders; and (3) good compliance during the 6-month monitoring course. Routine biochemical parameters, full peripheral blood counts, and routine urinalysis of the 436 eligible TB patients were examined monthly for 6 months or until the end of chemotherapy. Figure 2 shows a workflow diagram of the study. Five side effects to ATDs were assessed, including anemia, leukopenia, thrombocytopenia, drug-induced hepatotoxicity, and chronic renal damage. Anemia, leukopenia, and thrombocytopenia were established based on routine blood test results: hemoglobin concentration (Hb) ≤80 g/L, white blood cell count ≤2.0 × 10^9^/L, platelet count ≤75 × 10^9^/L, respectively [26]. Subjects with ATDH were diagnosed based on specialist consensus, as those patients with AST and/or ALT levels greater than three-times the maximum limit [27]. If eligible patients with TB had persistent kidney injury (e.g., hematuria, proteinuria, and casts) for longer than 90 days, they were diagnosed as having chronic kidney injury [28]. For each study subject, demographic and clinical information was extracted from the medical record system. We strictly obey the the Medical Association Declaration of Helsinki when using human tissue. This study was approved by the ethical committee of West China Hospital, Sichuan University, the relevant Judgement's reference number is NO. 198 (2014), and written informed consent was obtained from all the participants before blood sampling.

### Candidate SNP selection and genotyping assay

Candidate gene was selected based on their functional significance as key components of the Toll-liker receptor signaling pathway. Searching for International HapMap Project (http://www.hapmap.org/index.html.en), dbSNP database (http://www.ncbi.nlm.nih.gov/projects/SNP/) and 1000 Genomes database (http://www.1000genomes.org/), six candidate SNPs were identified for genotyping, according to the principles as follows: (1) SNPs were included if they were located in potential functional regions (exon, promoter and untranslated region); (2) SNPs could well represent those with minor allele frequency (MAF) >0.05 in a Chinese Han Beijing population; (3) we can work out the primers.

Blood samples from all participants were collected, and genomic DNA was extracted from EDTA anti-coagulated peripheral blood using the QIAamp DNA Blood Midi Kit (Qiagen, Germany) according to the manufacturer's protocol. The SNP genotyping work was performed using a custom-designed 2 × 48-Plex SNPscanTM Kit (Cat#:G0104, Genesky Biotechnologies Inc., Shanghai, China). Briefly, 100–200 ng of DNA was first denatured at 98°C for 5 min in a 10 μl reaction containing 1xDNA lysis buffer, and then mixed well with a 10 μl ligation premix composed of 2 μl 10x Ligase buffer, 0.5 μl Ligase, 1 μl Probe Mix and 7.5 μl Mili-Q water. The ligation reaction was done on an ABI 2720 thermocycler under the following cycling program: 4 cycles of 94°C for 1 min and 58°C for 4 hr, followed by 94°C for 2 min, and holding at 4°C. Reactions were immediately stopped by adding 20 μl of 2x Stop Buffer. Two 48-plex fluorescence PCR reactions were performed for each ligation product. Each PCR reaction was prepared in a 20 μl mixture containing 1xPCR Master Mix, 1 μl Primer Mix Set A or Set B and 1 μl ligation product. The PCR program was as follows: 95°C for 2 min; 9 cycles of 94°C for 20 s, 65°C–0.5°C/cycle for 40 s, 72°C for 1.5 min; 25 cycles of 94°C for 20 s, 57°C for 40 s, 72°C for 1.5 min; 60°C for 1 hr; followed by holding at 4°C. PCR products were separated and detected by capillary electrophoresis on an ABI3730XL sequencer. Raw data were analyzed by GeneMapper4.0, and genotypes for each locus were determined based on the information of the allele-specific ligation-PCR product labeling dye color and fragment size.

### Statistical analyses

SPSS software (version 19.0, SPSS Inc., USA), PLINK software (version 1.90) and Haploview software (version 4.2) were used to perform statistical analyses. The differences in general data and laboratory indices between the case group and control group were analyzed using Student's *t*-test, Chi-square test and Mann-Whitney *U*-test as appropriate. All genotype frequencies in the controls were evaluated for Hardy-Weinberg equilibrium. The distributions of allele, genotype and heritance model of selected SNPs within *TLR1* among cases and controls were evaluated by PLINK software. Linkage disequilibrium was assessed by calculating the pairwise *r*^2^ coefficient, and haplotypes were constructed with Haploview software. Possible associations among TB clinical and laboratory characterizations, ATD adverse reactions and *TLR1* SNPs were analyzed by employing the SPSS 19.0 software. Differences were considered significant when the *p* value <0.05.

## SUPPLEMENTARY MATERIALS FIGURES AND TABLES


