# Supplementary material for: TLR1 polymorphisms are significantly associated with the occurrence, presentation and drug-adverse reactions of tuberculosis in Western Chinese adults
Source: Oncotarget. 2017 Dec 8;9(2):1691–704. doi: 10.18632/oncotarget.23067 (PMC5788591; doi:10.18632/oncotarget.23067)
Supplement: Supplementary file 1 [file oncotarget-09-1691-s001.pdf]

TLR1 polymorphisms are significantly associated with the occurrence, presentation and drug-adverse reactions of tuberculosis in Western Chinese adults

SUPPLEMENTARY MATERIALS

Supplementary Table 1: Information of candidate 5 SNPs within *TLR1* gene

SNP	Chr: position	Amino acid change	MAF	HWE-P
Rs4833095	4:38798089	Asn→Ser,	0.41	0.171
Rs76600635	4:38798702	Ser→Pro,	0.09	0.724
Rs5743596	4:38800907	Ser→Leu	0.30	0.623
Rs5743565	4:38804362	none	0.43	0.583
Rs56357984	4:38804841	none	0.33	0.639
Rs5743557	4:38805206	none	0.43	0.903

Annotation: Chr: chromosome; HWE-P: *P* value of Hardy-Weinberg equilibrium tests in the control group.

Supplementary Table 2: Association of rs4833095 with ATDs adverse drug reactions

Drug adverse reactions	CT+TT (*n* = 280)	CC (*n* = 156)	*P*	OR (95% CI)
**Anemia *n* (%)**	75 (26.79)	32 (20.51)	0.145	1.42 (0.89–2.27)
**Leukopenia *n* (%)**	52 (18.57)	21 (13.46)	0.171	1.47 (0.85–2.54)
**Thrombocytopenia *n* (%)**	23 (8.21)	7 (4.49)	0.141	1.91 (0.80–4.55)
**Hepatotoxicity (%)**	32 (11.43)	13 (8.33)	0.309	1.42 (0.72–2.79)
**Chronic kidney damage *n* (%)**	61 (21.79)	33 (21.15)	0.878	1.04 (0.64–1.67)

Annotation: *p*-value was calculated using Chi-square test.

Supplementary Table 3: Association of rs56357984 with ATDs adverse drug reactions

Drug adverse reactions	AA+GA (*n* = 236)	GG (*n* = 200)	*P*	OR (95% CI)
**Anemia *n* (%)**	58 (24.58)	49 (24.50)	0.985	1.00 (0.65–1.56)
**Leukopenia *n* (%)**	34 (14.41)	39 (19.50)	0.156	0.70 (0.42–1.15)
**Thrombocytopenia *n* (%)**	16 (6.78)	14 (7.00)	0.928	0.97 (0.46–2.03)
**Hepatotoxicity (%)**	22 (9.32)	23 (11.50)	0.456	0.79 (0.43–1.47)
**Chronic kidney damage *n* (%)**	44 (18.64)	50 (25.00)	0.108	0.69 (0.43–1.09)

Annotation: *p*-value was calculated using Chi-square test.

Supplementary Table 4: Association of rs5743557 with ATDs adverse drug reactions

Drug adverse reactions	AA+AG (*n* = 284)	GG (*n* = 152)	*P*	OR (95% CI)
**Anemia *n* (%)**	66 (23.24)	41 (26.97)	0.388	0.82 (0.52–1.29)
**Leukopenia *n* (%)**	44 (15.49)	29 (19.08)	0.339	0.78 (0.46–1.30)
**Thrombocytopenia *n* (%)**	21 (7.39)	9 (5.92)	0.562	1.27 (0.57–2.84)
**Hepatotoxicity (%)**	25 (8.80)	20 (13.16)	0.154	0.64 (0.34–1.19)
**Chronic kidney damage *n* (%)**	57 (20.07)	37 (24.34)	0.301	0.78 (0.49–1.25)

Annotation: *p*-value was calculated using Chi-square test.

Supplementary Table 5: Association of rs5743596 with ATDs adverse drug reactions

Drug adverse reactions	AA+AG (*n* = 218)	GG (*n* = 218)	*P*	OR (95% CI)
**Anemia *n* (%)**	52 (23.85)	55 (25.23)	0.738	0.93 (0.60–1.44)
**Leukopenia *n* (%)**	31 (14.22)	42 (19.27)	0.158	0.70 (0.42–1.15)
**Thrombocytopenia *n* (%)**	12 (5.50)	18 (8.26)	0.256	0.65 (0.30–1.38)
**Hepatotoxicity (%)**	19 (8.72)	26 (11.93)	0.271	0.71 (0.38–1.32)
**Chronic kidney damage *n* (%)**	41 (18.81)	53 (24.31)	0.162	0.72 (0.46–1.14)

Annotation: *p*-value was calculated using Chi-square test.

Supplementary Table 6: Association of rs76600635 with ATDs adverse drug reactions

Drug adverse reactions	GG+AG (*n* = 75)	AA (*n* = 361)	*P*	OR (95% CI)
**Anemia *n* (%)**	13 (17.33)	94 (26.04)	0.111	0.60 (0.31–1.13)
**Leukopenia *n* (%)**	13 (17.33)	60 (16.62)	0.880	1.05 (0.54–2.03)
**Thrombocytopenia *n* (%)**	5 (6.67)	25 (6.93)	0.936	0.96 (0.36–2.59)
**Hepatotoxicity (%)**	5 (6.67)	40 (11.08)	0.253	0.57 (0.22–1.51)
**Chronic kidney damage *n* (%)**	16 (21.33)	78 (21.61)	0.958	0.98 (0.54–1.81)

Annotation: *p*-value was calculated using Chi-square test.
